# Crosstalk of Tumor-Derived Extracellular Vesicles with Immune Recipient Cells and Cancer Metastasis

**DOI:** 10.3390/cancers18020196

**Published:** 2026-01-07

**Authors:** Han Jie, Alicja C Gluszko, Theresa L. Whiteside

**Affiliations:** 1 Department of Pathology, UPMC Hillman Cancer Center, University of Pittsburgh School of Medicine, Pittsburgh, PA 15213, USA; hanxjx@upmc.edu (H.J.); gluszkoa@upmc.edu (A.C.G.); 2 Department of Biochemistry, Medical University of Warsaw, 1863 Warsaw, Poland; 3 Departments of Immunology and Otolaryngology, UPMC Hillman Cancer Center, University of Pittsburgh School of Medicine, Pittsburgh, PA 15213, USA

**Keywords:** tumor-derived extracellular vesicles, TEX, immune cells, cancer metastasis, HSP90/TLR2 crosstalk

## Abstract

**Background**. Contributions of tumor-derived extracellular vesicles, TEX, to tumor progression and metastasis involve their crosstalk with immune cells in the tumor microenvironment. This crosstalk results in metabolic reprogramming of immune cells from anti-tumor to pro-tumor activity. Mechanistic underpinnings of the TEX entry and delivery of molecular signals responsible for metabolic reprogramming may be unique for different types of immune cells. **Methods**. An in vitro model of THP-1 myeloid cells co-incubated with TEX illustrates the role TEX play in polarization of macrophages to TAMs. **Results**. In THP-1 cells, the dominant signaling pathway of melanoma cell-derived TEX involves HSP-90/TLR2. This leads to activation of the NF-κB and MAP kinase pathways and initiates THP-1 cell polarization from M0 to M2 with strong expression of immunosuppressive PD-L1. TEX may be seen as “danger” by the myeloid cells, which utilize the pattern recognition receptors (PRR), such as PAMPs or DAMPs, for engaging the complementary ligands carried by TEX. The same melanoma TEX signaling to T cells via DAMPs induced mitochondrial stress, resulting in T-cell apoptosis. **Conclusions**. As the signaling receptors/ligands in TEX are determined by the tumor, it appears that the tumor equips TEX with an address recognizing specific PRRs expressed on different recipient immune cells. Thus, TEX, acting like pathogens, are equipped by the tumor to alter the context of intercellular crosstalk and impose a distinct autophagy-not-apoptosis signature in recipient THP-1 cells. The tumor might endorse TEX to promote tumor progression and metastasis by enabling them to engage the signaling system normally used by immune cells for defense against pathogens.

## Simple Summary

The involvement of tumor-derived extracellular vesicles, TEX, in tumor progression and metastasis includes their crosstalk with various immune cells in the TME. An in vitro model of THP-1 myeloid cells co-incubated with TEX illustrates the role TEX play in polarization of macrophages to TAMs. TEX acting like DAMPs initiate the HSP-90/TLR2 axis signaling in THP-1 cells, activating the NF-κB and MAP kinase pathways and driving THP-1 cell polarization. This process of metabolic reprogramming of the immune cells from anti-tumor to pro-tumor activity promotes metastasis development.

## 1. Introduction

Tumor-derived extracellular vesicles (TEX) have been of special interest in the emerging field of immuno-oncology. This is largely due to the rapidly growing realization that TEX play a major role as promising cancer biomarkers on the one hand and, on the other, as carriers/distributors of molecular/genetic information between cells [1,2,3]. TEX are a heterogenous mix of small and large EVs produced by tumor cells and are present in body fluids of all cancer patients [3,4]. These tumor-derived EVs are morphologically indistinguishable from EVs produced and released by healthy cells, but their phenotypic, molecular and genetic features are different [5]. TEX mimic the molecular and genetic content of cancer cells and could be viewed as miniature editions of parental cancer cells [6].

Among various types of vesicles released by cancer cells, small extracellular vesicles (sEV) represent a subset of tumor cell-derived exosomes we call TEX that are sized at 30 to 150 nm and originate in the endocytic compartment of cancer cells [7]. Upon their release by the tumor cells into intercellular space, TEX migrate, enter the vascular and lymphatic systems and circulate freely reaching all tissues and all body fluids [8,9,10]. TEX have been extensively evaluated because they are surrogates of tumor cells and are responsible for cellular reprogramming of various cells in the tumor microenvironment (TME) [11]. TEX horizontally transfer their vesicular contents to the recipient non-malignant or malignant cells, altering their behavior. Current data suggest that TEX carry, transfer and deliver a broad variety of bioactive molecules, including proteins, lipids, glycans and nucleic acids localized in the vesicle lumen and on the vesicle surface [12]. TEX convert various immune cells in the tumor microenvironment (TME) from anti-tumor to pro-tumor activity, driving molecular/genetic events that may ultimately lead to tumor progression and metastasis [13].

Emerging evidence emphasizes the role of TEX as promoters of the pre-metastatic niche (PMN) formation [14]. TEX released from tumor tissues in large numbers move freely through body fluids interacting with all encountered cells and promoting distinct molecular events such as vascular leakiness, lymph angiogenesis, immune modulation, extracellular matrix remodeling, and metabolic reprogramming [10,15]. The inflammatory and immune systems responsible for the defense against foreign insults are primary targets a growing tumor wants to silence. TEX are delegated to perform the task of conversion of immune cells from anti-tumor effector cells to cells mediating pro-tumor functions. The mechanisms TEX utilize to accomplish the conversion are being extensively investigated because of their importance in PMN formation and metastasis development. The capability of TEX to engage, enter, and suppress anti-tumor activity of immune cells is directly linked to the initiation and promotion of the PMN formation as well as metastasis [15]. TEX pro-metastatic effects may be local and systemic [16], and likely contribute to the paucity of fully competent immune effector cells in situ as well as in the peripheral circulation of patients with cancer [17].

The interactions of TEX with immune cells that initiate their conversion to pro-tumor activity and lead to the PMN formation and metastasis are not clearly mapped and are under intense scrutiny. Here, we examine mechanisms TEX utilize for entry and reprogramming of THP-1 recipient cells used as surrogates for human macrophages. We report that TEX produced by melanoma cells and interacting with THP-1 cells are perceived as “DANGER SIGNALS” by these recipient cells. TEX engage pattern recognition receptors (PRRs) on the surface of recipient THP-1 cells and induce activation of the HSP-90/TLR2 signaling pathway. Further, the engagement by TEX of this dominant molecular pathway in THP-1 cells results in their functional reprogramming and promotes tumor progression.

## 2. Materials and Methods

**Cell lines**. Human melanoma cell line Mel526 derived from human uveal melanoma and THP-1, a human monocytic leukemia cell line, were purchased from the ATCC (American Tissue Culture Collection) and cultured in RPMI-1640 medium containing penicillin (100 units/mL) and streptomycin (100 µg/mL) and 10% (*v*/*v*) heat-inactivated fetal bovine serum (FBS, ThermoFisher Scientific, Waltham, MA, USA). The FBS was previously depleted of extracellular vesicles (EVs) by ultracentrifugation at 100,000× *g* for 3 h. Cells were cultured at 37 °C in an atmosphere of 5% CO_2_ in air, were routinely tested for Mycoplasma and were found to be mycoplasma free.

**Scheme 1:**
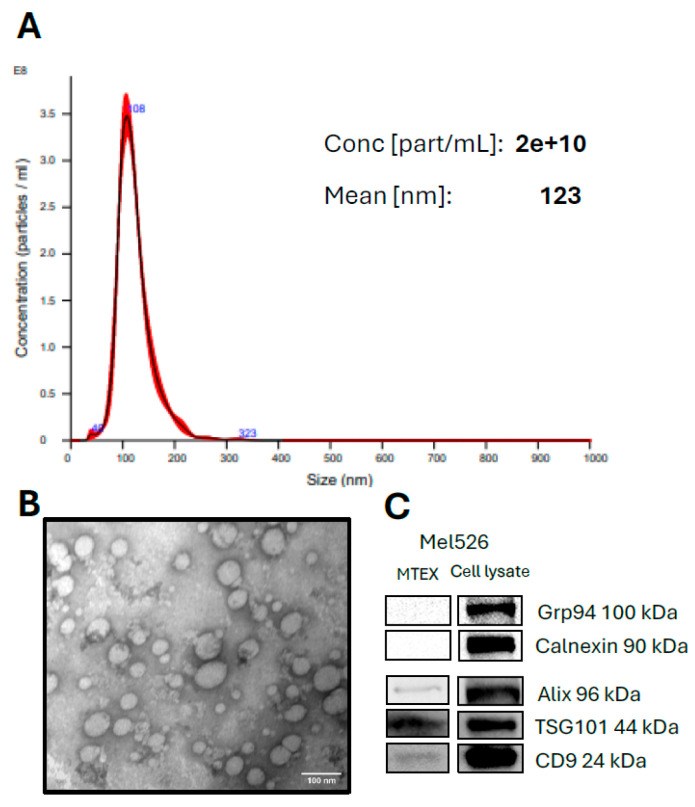
Characterization of MTEX in a representative fraction #4 isolated by SEC from supernatants of Mel 526 cells. In (**A**), NTA; In (**B**), TEM; and in (**C**), Western blots indicating the presence of ALIX, TSG101, CD9 in MTEX. Grp94 and calnexin are present in the cell lysate but are absent in MTEX.

**Isolation of melanoma cell-derived extracellular vesicles (MTEX).** Mel526 cells were cultured in 150 cm^2^ cell culture flasks containing 25 mL of culture medium. Each flask was seeded with 4 × 10^6^ cells, and following 72 h of incubation, supernatants were collected, while the cells were harvested using 2 mL of TrypLE Express (Gibco, Grand Island, NY, USA) and washed in serum-containing medium. For subsequent passages, cells were re-seeded in new flasks using the cell numbers described above. Supernatants were collected for isolation of MTEX as previously described [18] and were concentrated to 1 mL by using Vivacell 100 concentrators (Sartorius Corporation, Stonehouse, UK) at 2000× *g*. MTEX were isolated by size exclusion chromatography (SEC) as previously described [18,19] and as listed in EV-TRACK (ID = EV160007). Briefly, a 1 mL aliquot of the concentrated supernatant was loaded onto a 6 cm-long Sepharose 2B column (bed vol = 10 mL) and eluted with PBS at pH 7.0 (flow rate = 1 mL/min). Individual 1 mL fractions were collected. Fraction #4 containing the bulk of non-aggregated morphologically intact sEV [19] was harvested, concentrated using 100,000 MWCO Amicon filters (Merck Millipore Ltd, Tullagreen, Ireland.) and characterized as shown in Scheme 1.

### Methods Used for Characterization of MTEX Produced by Mel526 Cells

For **transmission electron microscopy** (TEM), freshly isolated sEV were placed on copper grids coated with 0.125% Formvar in chloroform and stained with 1% (*v*/*v*) uranyl acetate in ddH_2_O. TEM of sEV was performed at the Center for Biologic Imaging, the University of Pittsburgh, as previously described [19]. A JEM 1011 microscope was used for sEV visualization.

To measure **sEV concentration and size**, nanoparticle tracking analysis (NTA) with NanoSight 300 (Malvern, UK) was used, as previously described [19]. To determine the mean particle size/concentration in each sample, five consecutive measurements were obtained and averaged.

**Protein concentrations of sEV** were determined using a BCA protein assay (Pierce Biotechnology, Rockford, IL, USA) according to the manufacturer’s instructions. 

**Relative EV purity** was estimated by calculating the ratio of EV numbers per mL/EV protein in ug/mL) for each sample.

**Western blots** were performed to show the presence in MTEX of tetraspanins, CD9, CD63, CD81and endocytic proteins, ALIX and TSG101, and the absence of the cytosolic proteins, calnexin and grp94. Vesicle aliquots (10 µg protein) were lysed in Laemmli sample buffer (Bio-Rad, Hercules, CA, USA), separated using 4–15% SDS/PAGE gels and after transfer from gels to the polyvinylidene fluoride (PVDF) membranes, proteins were detected using Abs specific for antigens carried by sEV.

**Uptake of MTEX by THP-1 cells.** MTEX isolated SEC from supernatants of MEL526 cells were labeled with the MemGlow ^TM^ 590 dye (Cat. # MG03, Cytoskeleton, Inc., Denver CO, USA). Briefly, an aliquot of MTEX (30 µg) in 100 µL PBS was mixed with 0.5 µL of 20 µM MemGlow^TM^ 590 stock and incubated for 10 min in the dark at RT. Labeled vesicles were washed x1with PBS, as suggested by the dye manufacturer, using 100,000 MWCO Amicon filters (Merck Millipore Ltd.) and resuspended in 100 µL PBS. For uptake, 20 µL of freshly labeled MTEX were co-incubated with 2 × 10^4^ THP-1 cells in 100 µL RPMI for 0, 1, 2, 5, 10 and 15 min at 37 °C. Negative controls included cells treated with the dye only. Cells were washed with PBS, and MTEX uptake was visualized by confocal microscopy using x40 objective and quantified using a CytoFLEX flow cytometer (Beckman Coulter, Brea, CA, USA).

**Scheme 2:**
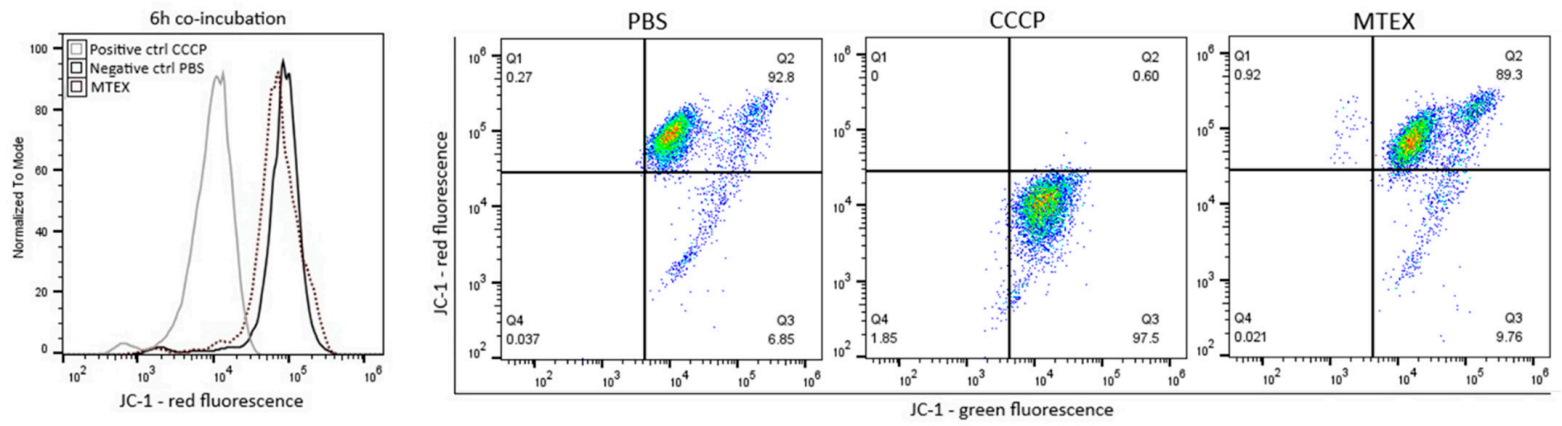
Results of the MMP assay with THP-1 cells co-incubated with MTEX, PBS (negative control), or CCCP (positive control) for 6 h. No mitochondrial membrane polarization by MTEX was observed in TPH-1 recipient cells after 6 h or 24 h co-incubation. Results are from 1/23 experiments performed.

**MTEX induced polarization of THP-1 cells.** THP-1 cells were seeded at 1 × 10^5^ cells/well in a 96-well plate and were differentiated into M0 macrophages by incubation with 150 nM phorbol 12-myristate 13-acetate (PMA, Sigma, P8139) for 24 h, followed by 24 h incubation in 100 µL of RPMI medium. Following this, THP-1 cells were tested by flow cytometry for expression levels of CD11b and CD68. THP-1 cells constitutively express CD11b and upregulated only CD68 after PMA treatment (Scheme 2). M0 macrophages were polarized to M1 type by incubation with 20 ng/mL of IFN-γ (ThermoFisher Scientific, Waltham, MA, USA: 485-MI-100) and 10 ng/mL of LPS (Sigma, Burlington, MA, USA, #L2880). Polarization of M0 to M2 macrophages was performed by incubation of M0 cells with 20 ng/mL of interleukin 4 (BioLegend, San Diego, CA, USA #574306) and 20 ng/mL of interleukin 13 (BioLegend, #571109) for 6 h. Macrophage polarization and PDL1 expression were measured after 6 h of co-incubation with 10 µg/mL of MTEX. The following Abs were used for staining: anti-human CD80-FITC (Beckman Coulter, #PN IM1853U), Mouse IgG1-FITC (PharMingen, San Diego, CA, USA #13854X), rat-antihuman IL-10-PE (eBioscience, San Diego, CA, USA #559330), Mouse IgG1-PE (eBioscience, #555749), anti-human CD274-eFluor450 (eBioscience, #48-5983-42), Mouse IgG1κ-eFluor450 (eBioscience, #48-4714-82). For staining, cells were suspended in the staining buffer (PBS + 3% BSA). Next, cells were blocked with anti-FcR reagent for 10 min at RT and then were incubated with pre-titered fluorochrome-labeled antibodies for 45 min at RT and with appropriate isotype controls. After washing in flow cytometry buffer, cells were immediately analyzed using a CytoFLEX flow cytometer (Beckman Coulter).

**Mitochondrial membrane potential assay.** To study MMP in THP-1 cells, the MitoProbeTM JC-1 Assay Kit (ThermoFisher Scientific, Cat. M34152) was used as per the manufacturer’s recommendations. Briefly, cultured TPH-1 cells were plated in wells of a U-bottom 96-well plate, at the concentration of 2 × 10^4^ cells/well in 100 µL RPMI medium. MTEX (10 µg/mL) were added to each well and co-incubated for 6 h. An aliquot (50 µM) of carbonyl cyanide 3-chlorophenylhydrazone (CCCP) was added to the cells as positive control and PBS was used as negative control. Following incubation, mitochondrial depolarization, which is indicated by a decrease in the red/green fluorescence intensity ratio, was measured by flow cytometry in a CytoFLEX flow cytometer (Beckman Coulter).

**Subcellular fractionation of THP-1 cells into mitochondria and cytosol.** THP-1 cells (2 × 10^6^/well) plated in wells of 6-well plates were co-incubated with TEX (10 µg/mL) isolated from supernatants of Mel526 or with PBS as control for 2, 4, 6 h as described above. The cells were harvested and sub-fractionated as previously reported into the mitochondrial and cytosol fractions [20]. Briefly, the cells were suspended in digitonin permeabilization buffer (50 mmol/L sucrose, 137 mmol/L NaCL, 70 mmol/L KCL, 4.3 mmol/L Na2HPO4, 1.4 mmol/L K2HPO4, 0.2 mg/mL digitonin and 0.1% hydroxylamine, vortexed and incubated on ice for 5 min. The cells were then centrifuged at 1000× *g* for 5 min at 4 °C. The supernatant contained the cytosolic fraction. The remaining pellet was resuspended in RIPA buffer (50 mmol/L Tris-HCL (pH 7.4), 150 mmol/L NaCL, 1 mmol/L EDTA, 1 mmol/L EGTA, 1% Triton X-100, 1% sodium deoxycholate and 0.1% SDS), vortexed and incubated on ice for 5 min. The lysate was vortexed and centrifuged at 10,000× *g* for 10 min at 4 °C. The supernatant contained the mitochondrial fraction. The subcellular fractions were resolved by SDS-PAGE, and the selected pro- or anti-apoptotic proteins were detected by immunoblotting. β-Actin and Cox IV served as controls for equal loading for the cytosol and mitochondrial fractions, respectively.

**Western blots of cell lysates, mitochondrial supernatants or cytosol.** For WB analyses, THP-1 cells (1 × 10^6^/mL/well) were co-incubated with Mel526 EVs (10 µg protein) or PBS as control. In selected experiments, anti-TLR2 Ab (0.5 µg/mL; Cell Signaling, Boston, MA, USA 12276) or Bafilomycin A1 (BafA1, 10 nM, Sigma, 131793) were added to wells containing THP-1 cells and EVs. Proteins in cell lysates, cell extracts, mitochondria, mitochondrial supernatants, or cytosol were resolved by SDS-PAGE and transferred to polyvinylidene difluoride membranes as previously described [20]. Following probing with a specific primary Ab and horseradish peroxidase-conjugated secondary Ab, the protein bands were detected by enhanced chemiluminescence (Pierce) and semi-quantitated in pixels using a UV Scan-IT software. The antibodies and Ab dilutions used for WBs are listed in Table S1.

**Statistical analysis.** Statistical analyses were performed using GraphPad Prism 10.0. All data sets were represented as the means ± SD obtained from three independent experiments. Statistical significance for the polarization experiments and mitochondrial membrane potential assays (based on mean fluorescence intensity measurements) was determined by comparing MTEX-treated cells with PBS-treated (negative control) cells using an unpaired two-tailed Student’s *t*-test. A *p*-value < 0.05 was considered statistically significant.

## 3. Results

**Characteristics of MTEX isolated from supernatants of Mel526 cells.** Small extracellular vesicles (sEVs) were isolated by size exclusion chromatography (SEC) as described in Methods from supernatants of Mel526 cells and evaluated for size (NTA), morphology (TEM), endocytic origin, and the absence of cytosolic proteins (WBs), as illustrated in Scheme 1. The mean size of EVs for 10 different EV samples was 129 nm. The relative purity of these EVs calculated as the ratio EV No/EV protein conc ranged from log8–9. The mean EV recovery was 2.8 × 10^11^/mL supernatant Based on these vesicle characteristics (size, biogenesis) and the ISEV criteria, they were classified as small extracellular vesicles (sEV) and, since they are a product of melanoma cells, are referred to as MTEX.

**Figure 1:**
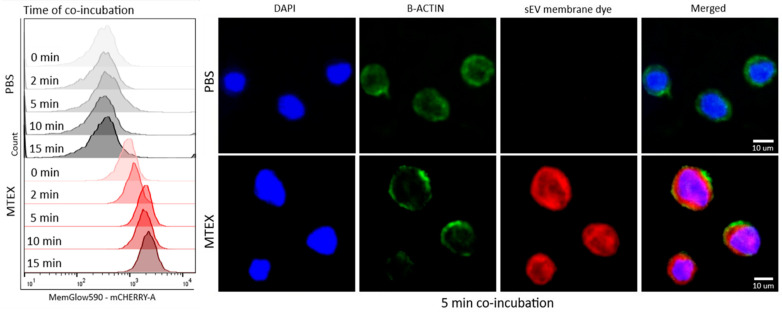
Uptake of MTEX labeled with the MemGlowTM 590 dye by THP-1 cells. Flow cytometry (**left panel**) and confocal microscopy (**right panel**) show a rapid entry of MTEX into the cytosol of THP-1 cells.

**Uptake of MTEX by THP-1 cells.** Interactions of tumor cell-derived vesicles with malignant or non-malignant recipient cells are the starting point of MTEX-driven reprogramming. The uptake of MTEX by THP-1 cells, as visualized by confocal microscopy and flow cytometry, is very rapid: within a minute of cell contact, a mass of labeled MTEX is seen in the cytoplasm of THP-1 cells (Figure 1). This type of massive and rapid vesicle entry suggests that phagocytosis or endocytosis are the responsible mechanisms. Further, within the next 5 min, labeled MTEX are seen in perinuclear location, consistent with their downstream processing. MTEX signaling at the surface of THP-1 cells, while brief, is nevertheless sufficient to initiate a series of downstream molecular and functional alterations in THP1 recipient cells.

**Signaling of MTEX interacting with THP-1 cells.** Recent data suggest that EVs in recipient cells interact with pattern recognition receptors (PRRs) located on the surface of immune recipient cells [21].

**Figure 2:**
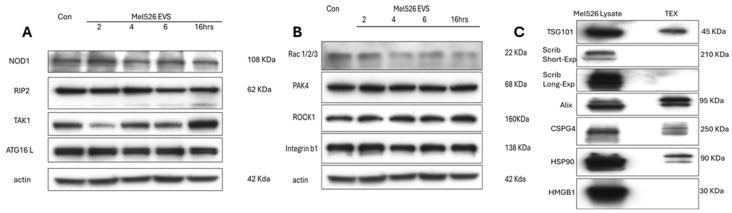
Changes in expression levels of various proteins in THP-1 cells following co-incubation with MTEX for various time periods. In (**A**), expression levels of NOD1-related proteins in THP1 cells. In (**B**), expression levels of Rho GTPase associated proteins and of Integrin β1 in THP1cells. In (**C**), comparative expression of various proteins detectable in melanoma cell lysate and in the isolated MTEX. Quantifications of WBs shown in (**A**,**B**) are in Table S1A.

**Figure 3:**
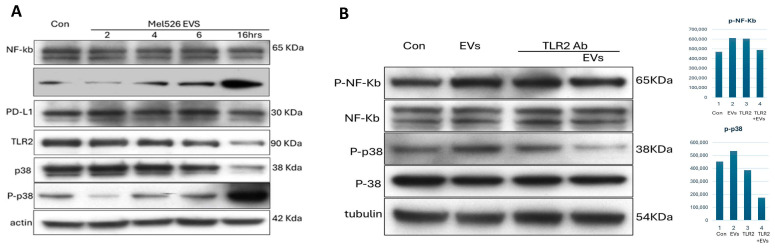
Activation of the NF-κb pathway by MTEX in THP-1 recipient cells. In (**A**), NF-κb phosphorylation, p38 phosphorylation and a decrease in expression levels of TLR2 in THP-1 cells co-incubated with MTEX. In (**B**), Expression levels of p-NF-κb and p-p38 are decreased in the presence of anti-TLR2 mAb (**right**).

It has been suggested that NOD1, the PRR recognizing the bacterial peptidoglycans, serves as a mediator of TEX driven polarization of macrophages [22]. Therefore, we co-incubated THP-1 cells (1 × 10^6^ per well) with MTEX (10 µg/mL) and tested THP-1 cell lysates for activation of the NOD1 pathway by WBs after 2, 4, 6, and 16 h of co-incubation. As shown in Figure 2A, NOD and RIP2 expression levels were slightly decreased after prolonged MTEX signaling suggesting only mild activation of the NOD1 pathway, with an increase in the expression level of the downstream TAK1. No change in expression levels of ATG16L was observed in THP-1 cells, indicating that MTEX signaling via NOD did not activate autophagy in these cells. Since NOD is known to be responsive to Rho GTPase activation states [23], we asked whether MTEX induced Rho GTPase was signaling in THP-1 cells. Figure 2B shows no evidence for the GTPase pathway activation in response to MTEX-mediated signaling, as expression levels of Rac1/2/3, PAK4, ROCk1 and integrin-β1 were not altered even after prolonged exposure to MTEX. This suggested that MTEX did not carry factors which activate small Rho-GTPases in THP-1 cells. Since SCRIB is a known Rho-GTPase activator [24], we searched for its expression on MTEX and found it absent, although the melanoma cell lysate was rich in SCRIB (Figure 2C). These data ruled out the NOD pathway as the recipient of MTEX signaling in THP-1 cells. Interestingly, another PRR, the HSP90, a chaperone protein and a well-known promoter of inflammatory responses was carried by MTEX (Figure 2C). Its cognate receptor TLR2 was expressed on THP-1 cells and utilized, as indicated by the declining level of its expression over time (Figure 3A). Concomitantly with the decreased TLR2 protein expression at 16 h of co-incubation, an intense phosphorylation of p38 and equally intense phosphorylation of the NF-κB were observed, indicating activation by MTEX of the MAPK pathway as well as the NF-κB pathway (Figure 3A). The concomitant phosphorylation of p38 and NFκB, indicating activation of both these pathways, significantly contributes to inflammatory gene transcription. In aggregate, binding of HSP90 carried by MTEX to TLR2 expressed in THP-1 cells initiates activation of the cascade of proteins that leads to the NF-κB and MAP kinase activation and culminates in transcription of inflammatory genes, driving polarization of THP-1 cells. It is interesting to note that although MEL526 melanoma cells contained HMGB1, a potent activator of inflammatory responses, MTEX did not carry this protein.

**MTEX signaling in THP-1 cells induces autophagy but not intrinsic apoptosis.** As shown above, signaling initiated by MTEX is interpreted as “danger” by THP-1cells and leads to activation of the HSP-90/TLR2 pathway, one of the classical “danger associated molecular patterns” (DAMPS) that induce alterations in functions of the recipient cells. Based on our previous data suggesting that MTEX-driven DAMPS signaling in T cells leads to mitochondrial dysfunction and intrinsic T cell apoptosis [1], we expected to see similar results in THP-1 recipient cells. However, the initial MMP assay results with THP-1 cells co-incubated with MTEX indicated minimal disturbance of the mitochondrial functions (Scheme 2).

**Figure 4:**
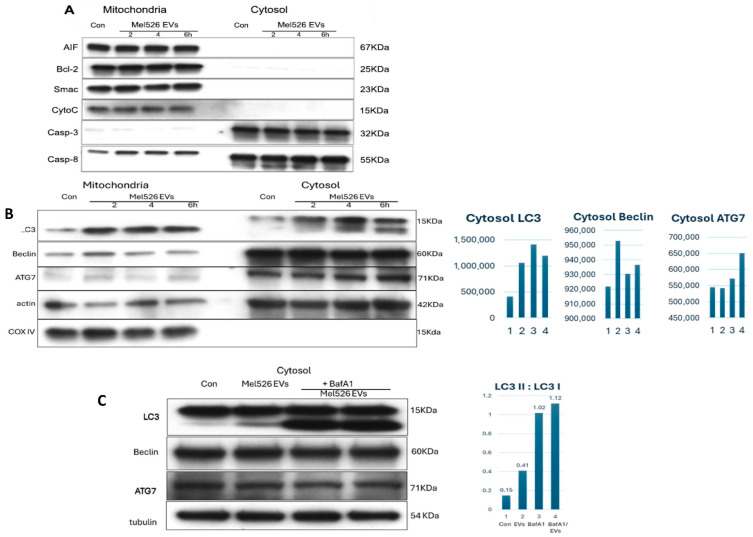
MTEX co-incubated with THP-1 cells did not induce mitochondrial dysfunction (**A**). Instead, MTEX signaling induced accumulation of the autophagy associated proteins, LC3 and Beclin, in the cytosol (**B**). In the presence of Bafilomycin A1, the LC3 II expression increased as did the LC3II/LC3I ratio (**C**), (**right**).

When the THP-1 cells co-incubated with MTEX were fractionated into the mitochondrial and cytosol fractions, no evidence for leakage of mitochondrial proteins, AIF, Smac, Cytochrome C, Bcl-2, into the cytosol were seen, and no cleavage of caspase 3 and 8 occurred (Figure 4A). These results indicated that MTEX signaling in THP-1 did not result in intrinsic apoptosis of the recipient cells. Instead, MTEX induced accumulation of autophagy-related proteins, LC3, Beclin, ATG7 in the cytosol, was consistent with the autophagic flux (Figure 4B). Importantly, in the presence of Bafilomycin A1, which disrupts autophagic flux, accumulation of LC3II in the THP-1 cytosol was evident (Figure 4C).

**Figure 5:**
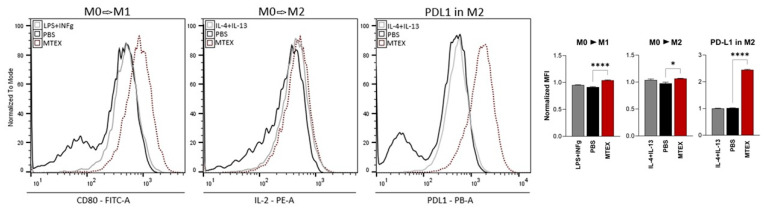
Polarization of TPH-1 cells from M0 to M1 and M0 to M2 following co-incubation in the presence of MTEX relative to LPS or cytokine-induced polarization. High level of PD-L1 expression induced by MTEX is consistent with the M2 phenotype of THP-1 cells. Shown is the representative experiment of two performed. * *p* < 0.05; **** *p* < 0.0001.

**Functional effects of MTEX uptake by THP1 cells**. Various reports in the literature suggest that tumor-derived EVs promote macrophage polarization [25,26,27]. We have co-incubated THP-1 cells with MTEX and observed the conversion of M0 to M1 and M0 to M2 in the recipient cells Figure 5. 

## 4. Discussion

The involvement of TEX in cancer progression and metastasis has been long recognized [10,13,14] and has been extensively evaluated in the last decade [15]. The ability of TEX to induce, promote and regulate metastasis was commented upon recently, and the TEX active role in establishing the pre-metastatic niche (PMN) formation has been emphasized [26,28,29]. This function of TEX fits well with their overall responsibility of mediating crosstalk between the tumor and non-malignant cells within its milieu. In fact, TEX-mediated reprogramming of immune cells, including dendritic cells (DCs), neutrophils, macrophages, natural killer (NK) cells, and various subsets of T cells emerges as one of the main mechanisms that contribute to the PMN formation [26].

Tumors produce an excess of TEX which circulate freely and thus have access to circulating and tissue infiltrating immune cells [9]. Upon initiating contacts with immune cells, TEX simultaneously deliver multiple signals to recipient immune cells, and it is likely that the quality and quantity of TEX-mediated signaling regulates the cellular responses. The vesicle entry into a recipient cell involves at least two separate events: (i) signaling by receptors situated on the recipient cell surface membrane and (ii) a loss of vesicular morphology in the cytosol and delivery of the vesicular cargo, including proteins, lipids, glycans, and nucleic acids, that initiates a wave of signaling resulting in metabolic reprogramming of recipient cells. The MTEX lipo-protein surface membrane is decorated by numerous cancer cell-derived receptors and ligands [17], which are expected to interact with complementary molecules on the immune cell surface, initiating the tumor-recipient cell crosstalk. Prominent among these receptor-ligand complexes are pattern recognition receptors (PRRs), such as PAMPs or DAMPs, which recognize extracellular pathogens or components of damaged cells, respectively [21]. It appears that MTEX, emulating pathogens, utilize the same mechanisms of signaling their presence at the THP-1 cell surface. Interestingly, it is TLR2 in THP-1 cells, a well-known PAMP, that upon recognizing HSP-90 in MTEX signals “danger” to cellular machinery. Surprisingly, HMGB1, a common PRR present in lysates of the parent melanoma cells, was not detected on the surface of MTEX. This suggests that the tumor might reserve the option of selecting the proteins used for vesicular interactions with recipient cells, thereby providing “an address” for vesicular activity that in the case of THP-1 recipient cells involved HSP-90 rather than HMGB1. It is possible that TEX interacting with different types of immune cells deliver distinct signals that drive subsequent reprogramming through different molecular events resulting in altered functional profiles.

In the experiments reported here, a melanoma cell line, MEL526, was the source of MTEX co-incubated with THP-1 cells. The same MTEX were previously used by us to investigate signaling induced by MTEX in CD8+Jurkat T cells [1]. We observed that MTEX-induced signaling in activated CD8+ T cells involved the engagement of DAMPs on the T cell surface. This was followed by activation of stress-associated molecular pathways, metabolic rewiring of T cells via mitochondrial dysfunction culminating in apoptosis or autophagy [1]. In THP-1 cells the same MTEX-mediated signaling involved the HSP-90/TLR2 crosstalk. TLR2 is a well-known PRR, and its engagement by MTEX has led to activation of the NF-κB pathway that promoted THP-1 polarization from M1 to M2 with the upregulated PD-L1 expression. These polarized macrophages are expected to support tumor growth and mediate immune suppression [30]. In considering TEX abilities to reprogram different types of immune cells, it is interesting to observe that the MTEX originating from supernatants of the same cultured MEL526 cells and presumably bearing similar molecular profiles can utilize distinct signaling and reprogramming mechanisms when interacting with macrophages vs. lymphocytes. This suggests that the tumor and TEX it produces determine the outcome of the interaction, and that TEX, like pathogens, engage PAMPs variously expressed on different cells to enter and initiate molecular reprogramming.

In this study, THP-1 leukemia monocytic cells are used as surrogates for primary human macrophages. Thus MTEX-induced signaling and polarization effects observed with THP1 cells may differ from those MTEX induce in primary monocytes/macrophages. Clearly, a validation of the reported data with PBMC-derived or tissue-derived monocytes/macrophages will be required. Nevertheless, our earlier studies of glioblastoma-derived TEX interacting with primary monocytes or macrophages provide support for our current conclusions. The glioblastoma-derived TEX activated the NF-κB pathway and promoted cellular differentiation from M0 to M1 (mediate pro-inflammatory functions) to M2 (mediate anti-inflammatory functions) in primary monocytes/macrophages both in vitro and in vivo [31]. As a result of this M2-like polarization driven by TEX, macrophages acquired pro-tumor functions that characterize tumor-associated macrophages (TAMs): they produced and secreted small EVs rich in Arginase-1, which, upon co-incubation with tumor cells, promoted tumor growth [30]. Interestingly, while macrophages co-incubated with glioblastoma-derived TEX showed high phosphorylation levels of MAPK1,2; ERK1,2; p38a; c-Jun, HSP-27 and CREB, a different set of proteins were phosphorylated in CD8+ T cells co-incubated with the same TEX [31]. These results suggested that glioblastoma TEX induced activation of distinct molecular pathways in macrophages vs. T lymphocytes, culminating in polarization of the former and in apoptosis in the latter [31]. Together with the results of MTEX signaling in THP-1 recipient sells reported here, it appears that a “division of labor” among TEX in selecting the actionable target for reprogramming originates with the tumor.

## 5. Conclusions

In the context of intercellular communication, the crosstalk between tumor and non-malignant cells in the TME is driven by TEX emerges as the major contributor to the metabolic conversion and reprogramming of immune cells from anti-tumor to pro-tumor activity. Thus, TEX play a major role in promoting tumor progression and metastasis, and this role appears to be imposed on TEX by the tumor. The development of strategies for elimination or silencing of TEX that are programmed by tumor to mediate pro-tumor effects represents an urgent and highly challenging therapeutic objective for the medical community.

## Data Availability

All relevant data used to conduct the analyses are available within the manuscript. Supplementary Data for this article are available online. All additional information can be obtained by contacting the senior author.

## Supplementary Materials

The following supporting information can be downloaded at: https://www.mdpi.com/article/10.3390/cancers18020196/s1, Table S1A: THP-1 co-incubated with Mel526 EVs: WB quantification for Figure 2A,B; Table S1B: THP-1 co-incubated with Mel526 EVs: WB quantification for Figure 3A,B.
