# Supplementary material for: Crosstalk of Tumor-Derived Extracellular Vesicles with Immune Recipient Cells and Cancer Metastasis
Source: Cancers (Basel). 2026 Jan 7;18(2):196. doi: 10.3390/cancers18020196 (PMC12839004; doi:10.3390/cancers18020196)
Supplement: Supplementary file 1 [file cancers-18-00196-s001.zip › cancers-4021918-supplementary.pdf]

TABLE S1A. THP-1 co-incubated with Mel526 EVs: WB quantification for Figures 2A and 2B.

Quantification was performed by normalizing intensity of the band of interest against intensity of actin loaded as control

Fig2A	NOD1	Ratios to actin	RIP2	Ratios to actin	TAK1	Ratios to actin	ATG16L	Ratios to actin
	Con	0.479	Con	1.385	Con	0.053	Con	1.113
	Evs 2hr	0.546	Evs 2hr	1.286	Evs 2hr	0.065	Evs 2hr	1.056
	Evs 4hr	0.277	Evs 4hr	1.316	Evs 4hr	0.134	Evs 4hr	0.899
	Evs 6hr	0.388	Evs 6hr	0.998	Evs 6hr	0.114	Evs 6hr	0.965
	Evs 16hr	0.235	Evs 16hr	1.163	Evs 16hr	0.161	Evs 16hr	0.897

Fig2B	Rac 1/2/3	Ratios to actin	PAK4	Ratios to actin	Rack1	Ratios to actin	Integrin b	Ratios to actin
	Con	0.258	Con	1.061	Con	0.693	Con	1.224
	Evs 2hr	0.171	Evs 2hr	0.984	Evs 2hr	0.732	Evs 2hr	1.261
	Evs 4hr	0.025	Evs 4hr	0.962	Evs 4hr	0.809	Evs 4hr	1.026
	Evs 6hr	0.088	Evs 6hr	0.905	Evs 6hr	0.744	Evs 6hr	1.071
	Evs 16hr	0.058	Evs 16hr	0.978	Evs 16hr	0.928	Evs 16hr	1.157

Table S1B**. THP-1 co-incubated with Mel526 EVs: WB quantification for Figures 3A and 3B.**

Quantification was performed by normalizing intensity of the band of interest against intensity of actin loaded as control

Fig3A	**NF-Kb**	**Ratios to actin**	**p-NF-Kb**	**Ratios to actin**	**PD-L1**	**Ratios to actin**	**TLR2**	**Ratios to actin**	**P38**	**Ratios to actin**	**p-p38**	**Ratios to actin**
	Con	0.974	Con	0.19	Con	1.287	Con	1.215	Con	1.265	Con	0.335
	Evs 2hr	0.895	Evs 2hr	0.064	Evs 2hr	1.532	Evs 2hr	1.097	Evs 2hr	1.342	Evs 2hr	0.135
	Evs 4hr	0.854	Evs 4hr	0.291	Evs 4hr	1.307	Evs 4hr	0.952	Evs 4hr	1.28	Evs 4hr	0.359
	Evs 6hr	0.893	Evs 6hr	0.464	Evs 6hr	1.499	Evs 6hr	0.74	Evs 6hr	1.021	Evs 6hr	0.578
	Evs 16hr	0.847	Evs 16hr	1.158	Evs 16hr	1.248	Evs 16hr	0.385	Evs 16hr	0.447	Evs 16hr	1.573

THP-1 co-incubated with Mel526 EVs in the presence of anti-and TLR2 Ab

Fig3B	**p-NF-Kb**	**Ratios to tubulin**	**NF-Kb**	**Ratios to tubulin**	**p-p38**	**Ratios to tubulin**	**p38**	**Ratios to tubulin**
	Con	0.783	Con	1.126	Con	0.758	Con	1.292
	Evs	1.091	Evs	1.214	Evs	0.953	Evs	1.269
	TLR2	0.936	TLR2	1.078	TLR2	0.6	TLR2	1.081
	TLR2, Evs	0.798	TLR2, Evs	1.213	TLR2, Evs	0.284	TLR2, Evs	1.036
